# Circulating and disseminated tumor cells in pancreatic cancer and their role in patient prognosis: a systematic review and meta-analysis

**DOI:** 10.18632/oncotarget.19928

**Published:** 2017-08-04

**Authors:** David Stephenson, Christopher Nahm, Terence Chua, Anthony Gill, Anubhav Mittal, Philip de Reuver, Jaswinder Samra

**Affiliations:** ^1^ Sydney Medical School, University of Sydney, Camperdown, Sydney, Australia; ^2^ Upper GI Surgical Unit, Department of Gastrointestinal Surgery, Royal North Shore Hospital, St. Leonards, Sydney, Australia; ^3^ Cancer Diagnosis and Pathology, Kolling Institute, Royal North Shore Hospital, St. Leonards, Sydney, Australia; ^4^ Department of Surgery, Radboud University Medical Centre, Nijmegen, The Netherlands

**Keywords:** circulating tumor cells, disseminated tumor cells, pancreatic cancer, prognosis

## Abstract

**Background:**

Disseminated tumor cells (DTCs) and circulating tumor cells (CTCs) have been postulated to seed metastases and contribute to poorer patient outcomes in many types of solid cancer. To date, no systematic reviews have examined the role of both DTCs and CTCs in pancreatic cancer. We aimed to determine the prognostic value of DTCs/CTCs in pancreatic cancer using a systematic review and meta-analysis.

**Materials and Methods:**

A comprehensive literature search identified studies examining DTCs and CTCs in the bone marrow and blood of pancreatic cancer patients at diagnosis with follow-up to determine disease-free/progression-free survival (DFS/PFS) and overall survival (OS). Statistical analyses were performed to determine the hazard ratio (HR) of DTCs/CTCs on DFS/PFS and OS.

**Results:**

The literature search identified 16 articles meeting the inclusion criteria. The meta-analysis demonstrated statistically significant HR differences in DFS/PFS (HR = 1.93, 95% CI 1.19–3.11, *P* = 0.007) and OS (HR = 1.84, 95% CI 1.37–2.45, *P* =< 0.0001), indicating patients with detectable DTCs/CTCs at diagnosis have worse prognoses. Subgroup analyses suggested CTCs in the peripheral blood (HR =2.03) were more indicative of poor OS prognosis than DTCs in the bone marrow (HR = 1.91), although the difference between these was not statistically significant. Positivity of the CellSearch detection method for DTC/CTC had the highest correlation with decreased OS (HR = 2.79) while immunodetection (HR = 1.91) and RT-PCR (HR = 1.25) were less effective in determining prognosis.

**Conclusion:**

The detection of DTCs/CTCs at diagnosis is associated with poorer DFS/PFS and OS in pancreatic cancer.

## INTRODUCTION

Pancreatic cancer (PC) is the fourth most common cause of cancer-related death in the United States, resulting in an estimated 41,000 deaths each year [1]. Globally, PC is the seventh most common cause of cancer-related death, resulting in the deaths of 173,800 males and 156,600 females annually [2]. Currently, the 5-year survival rate in the United States is 8% [1].

Due to the paucity of early detection methods for PC, there has been great interest in identifying novel biological markers. Circulating tumor cells (CTCs), tumor cells shed from the primary tumor circulating through the vascular system, have been proposed as a potential biological marker for early detection, prognosis, treatment selection and monitoring disease progression in PC [3–6]. Disseminated tumor cells (DTCs) are a subset of CTCs which have migrated to a new location where they can survive and potentially develop into metastases [7]. DTCs are frequently identified in the bone marrow (BM) of many types of solid carcinomas, acting as a reservoir of tumor cells which can then re-enter the circulation and spread to distant organs [8].

A variety of methods exist for CTC/DTC detection and identification. The three main methodologies are antibody-based detection methods, including the CellSearch® system (Veridex LLC), reverse transcription polymerase chain reaction (RT-PCR), and techniques developed to isolate CTCs/DTCs based upon their biological and physical properties allowing label-free isolation of viable CTCs/DTCs. To date, most studies have utilized immunological and RT-PCR based techniques. The prognostic value of CTCs has been examined in a variety of solid cancers, including a number of meta-analyses [9–30]. In most tumor types, there is a significant correlation between CTC-positivity and poorer survival outcomes, including overall survival (OS), disease-free survival (DFS), or progression-free survival (PFS). Recently, two meta-analyses examined the prognostic value of CTCs in PC [31, 32], also finding a correlation between CTC-positivity and worse survival outcomes. To date, there have not been any meta-analyses examining the prognostic role of DTCs in PC.

In the present study, we aimed to perform a systematic review and meta-analysis to identify and analyze the prognostic value of both CTCs and DTCs in PC. The study aimed to encompass all CTC/DTC detection methods which have been published in combination with survival data to provide a comprehensive analysis of all CTC/DTC detection methods and their utility in PC prognosis. This study is the first to perform a pooled analysis of the prognostic value of DTCs in PC.

## RESULTS

### Literature search

**Figure 1:**
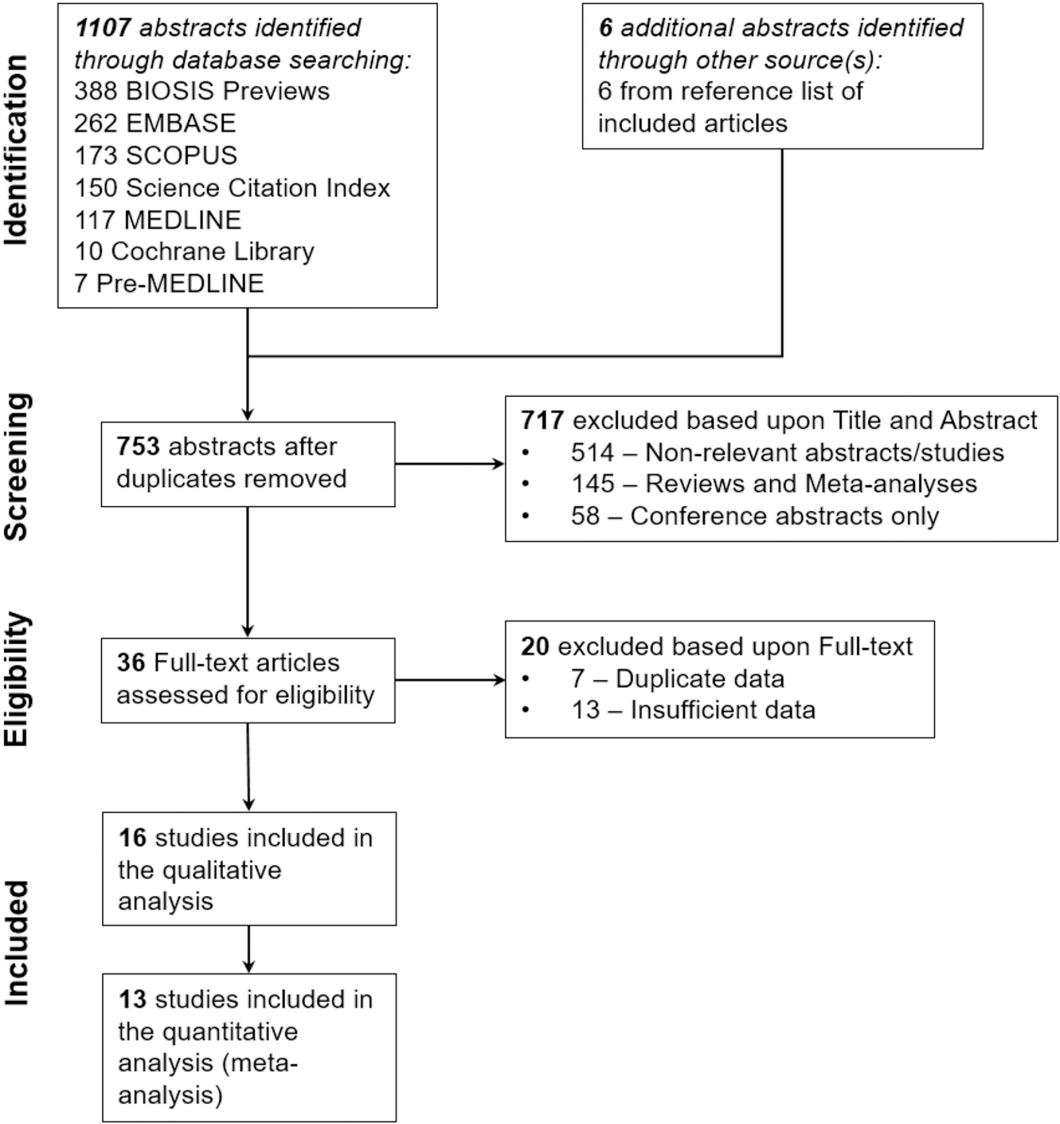
Flow diagram demonstrating the selection of studies for inclusion in this systematic review

The comprehensive literature search identified a combined total of 1109 articles, while four additional articles were identified from the reference list and cited article searches. Figure 1 illustrates the review process leading to the selection of the 16 included articles [33–48]. Following the removal of duplicates 753 unique articles were identified. Titles and abstracts were assessed for their relevance to this study and 717 articles were excluded. A further 20 articles were excluded upon examining the full text as they did not meet the inclusion criteria. Therefore, 16 articles were included for further analysis. Of these, 13 articles were analyzed using meta-analysis and systematic review, while three studies had insufficient data for the meta-analysis and were therefore only included in the systematic review.

### Study characteristics

**Figure 2:**
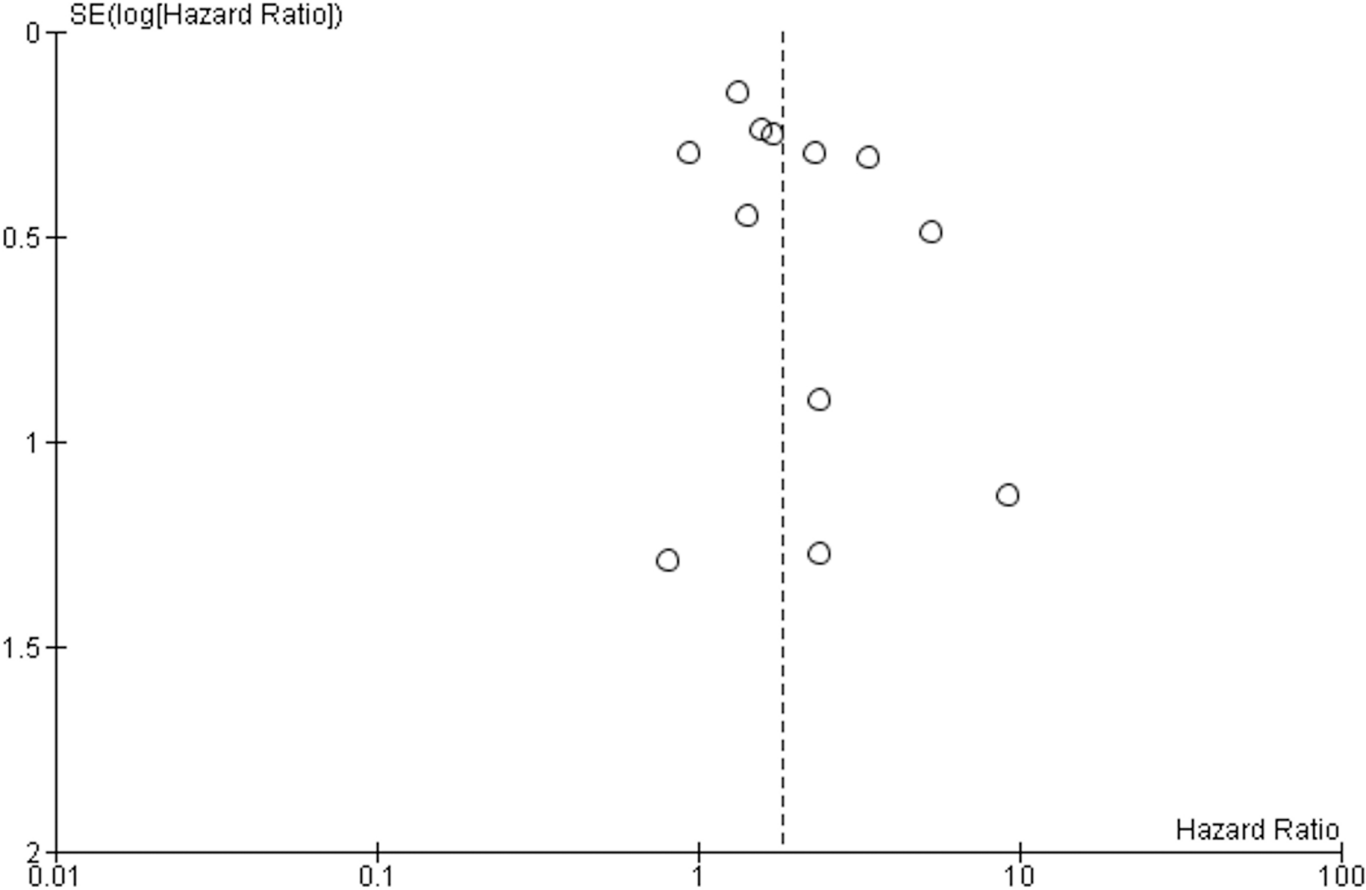
Funnel plot analysis of publication bias for OS studies

**Figure 3:**
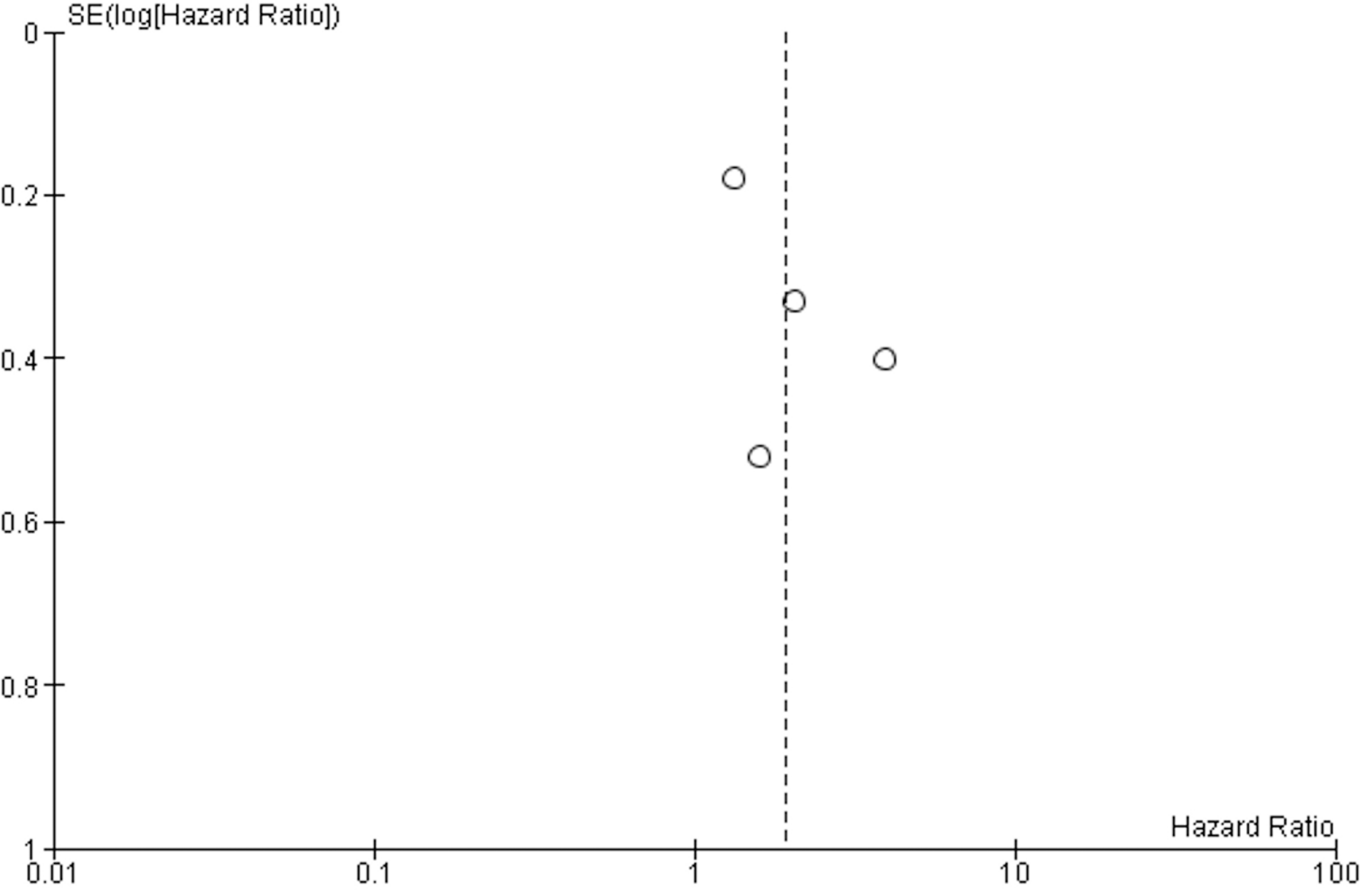
Funnel plot analysis of publication bias for DFS/PFS studies

The baseline characteristics of the studies included in this systematic review and meta-analysis are included in Table 1. The CTC/DTC status and survival data (OS,DFS and/or PFS) was analyzed in 855 PC patients (325 CTC/DTC-positive and 530 CTC/DTC-negative). The study design variables are summarized in Table 2. Samples were collected from the BM (8 studies), and PB (10 studies). Five studies [36, 38, 40, 44, 47] examined the presence/absence of CTC/DTC in peritoneal lavage samples, but the analysis of this data was beyond the scope of the current study. The detected CTC/DTC levels reported in the current study reflect only those detected in the BM and PB. Immunodetection (ID) (immunocytochemistry [ICC] and immunohistochemistry [IHC]) techniques (8 studies), RT-PCR (6 studies) and the commercial CellSearch method (3 studies) were used to detect CTCs/DTCs. Only one study reported data using multi-modal detection of CTCs/DTCs (CellSearch and ID) [39]. In all studies, samples for CTC/DTC analysis were taken prior to treatment for PC, and three of these studies also examined CTC/DTC levels after treatment [33, 36, 44]. Five studies examined the relationship between the detection of CTCs/DTCs and DFS/PFS [34, 35, 39, 42, 44]. All studies, except one [34], examined CTC/DTC positivity in relation to OS. Two studies performed multivariable analyses [33, 35]. The risk of bias within each individual study was assessed (Table 3) indicating 10 studies had low risk of bias [33–35, 37–39, 41, 44, 45, 47], while six studies were at high risk of bias [36, 40, 42, 43, 46, 48]. Funnel plot analyses were performed to assess for risk of bias across all studies (Figure 2 and Figure 3). The funnel plot analysis of the OS studies did not reveal any obvious asymmetry indicating there was not any relevant publication bias. The funnel plot analysis of the DFS/PFS studies only included four studies, making it more difficult to determine any publication bias. Despite this, there were no obvious outliers in the funnel plot.

**Table 1:** Baseline characteristics of the included studies of this systematic review

First author	Year	Country of origin	Sample size (M/F)	Age (range)		TNM classification scheme	TNM Stage	Palliative/curative intent	Treatment (surgery, chemotherapy, radiotherapy)	Positive CTC/DTC patients (%)^a^	CTC/DTC analyzed	
				Median	Mean							
Bidard [33]	2013	France	79 (34/45)	-	-	UICC 2002	III	Palliative	Chemotherapy	4 (5%)	CTC	
De Albuquerque [34]	2012	Germany	34 (20/14)	67 (55–74)	-	NR	II–IV	Both	Chemotherapy	16 (47%)	CTC	
Effenberger [35]	2012	Germany	175 (96/79)	67 (33–84)	-	UICC	I–IV	Both	NR	24 (14%)		DTC
Hoffmann [36] ^b^	2007	Germany	37 (-/-)	-	-	UICC 2005	I–IV	Both	Surgery	8 (22%)	CTC	DTC
Hu [37]	2013	China	46 (28/18)	-	58 (44–80)	NR	I–IV	Both	NR	41 (89%)	CTC	
Juhl [38]	1994	Germany	34 (19/15)	-	61 (46–78)	UICC 1987	I–IV	Both	Surgery	17 (61%)		DTC
Khoja [39]	2012	England	54 (29/25)	-	– (35–85)	NR	III–IV	Palliative	NR	21 (40%)	CTC	
Klos [40]	2010	Czech Republic	70 (42/28)	-	63	UICC	NR	Potentially curative	Surgery	22 (44%)	CTC	
Kurihara [41]	2008	Japan	26 (15/11)	70 (51–82)	-	NR	II–IV	Both	Chemotherapy ± surgery	11 (42%)	CTC	
Rehders [42] ^b^	2012	Germany	108 (61/47)	66 (41–85)	-	UICC 2002	I–IV	Potentially curative	Surgery and chemotherapy	12 (24%)		DTC
Roder [43]	1999	Germany	48 (23/25)	-	63 (53–73)	UICC 1992	I, III, IV	Both	Surgery and chemotherapy	25 (52%)		DTC
Sergeant [44]	2011	Belgium	40 (23/17)	-	-	AJCC 2010	I, II, IV	Both	Surgery	10 (25%)	CTC	
Soeth [45]	2005	Germany	172 (83/89)	-	-	UICC 1997	I–IV	Both	Surgery	81 (47%)	CTC	DTC
van Heek [46]	2001	Netherlands	35 (19/16)	63 (42–77)	-	UICC 1997	I–IV	Both	Surgery	10 (32%)		DTC
Vogel [47]	1999	Germany	80 (-/-)	-	-	UICC	I–IV	Both	Surgery	27 (38%)		DTC
Zhang [48] ^b^	2015	China	22 (10/12)	-	-	NR	I–IV	NR	Surgery ± chemo-radiotherapy	15 (68%)	CTC	

^a^Pre-treatment positive patients (before any intervention e.g. chemotherapy/surgery/palliation), ^b^Not included in meta-analysis studies.

*NR* not reported, *CTC* circulating tumor cell, *DTC* disseminated tumor cell, *M/F* male/female.

**Table 2:** Study design variables of the included studies of this systematic review

First author	Sampling site	Sampling time	Detection method	Target antigen/gene	Definition CTC/DTC positivity	CTC/DTC Detection rate (%)		Follow up		Hazard Ratio (HR) estimate (Tierney [78] method no.)	Outcome	Multivariable analysis
						Baseline	Overall	Mean (± SD)	Median (range)			
Bidard [33]	PB	Pre, post	CellSearch	EpCAM, CK (8, 18, 19), EGFR	≥ 1 cell	4/75 (5)	9/79 (11)	-	-	Estimated from K-M curve (10)	OS	Yes
De Albuquerque [34]	PB	Pre	RT-PCR	mucin 1, EpCAM, KRT19, MUC1, CEACAM5, BIRC5	≥ 1 mRNA marker amplified	16/34 (47)	-	-	12.5 (2–26)	Estimated from K-M curve (10)	PFS	No
Effenberger [35]	BM	Pre	ICC	CK (7, 8, 18)	≥ 1 cell	24/175 (14)	-	-	-	Estimated from K-M curve (10)	PFS, OS	Yes
Hoffmann [36]	PB	Pre, intra, post	RT-PCR	CK-19	> highest level in controls	8/37 (22)	-	-	-	Unable to estimate – insufficient data	OS	No
	BM					0/37 (0)						
Hu [37]	PB	Pre	RT-PCR	h-TERT	-	31/46 (67)	-	-	16.5 (6–30)	Estimated from K-M curve (10)	OS	No
				c-Met	-	41/46 (89)						
Juhl [38]	BM	Pre	IHC	CK (1, 2, 5, 6, 7, 8, 11, 14, 16, 17 ,18), mucin, CEA, Ca-19–9, 17–1A (membrane antigen)	-	17/28 (61)	-	-	-	Estimated from K-M curve (10)	OS	No
Khoja [39]	PB	Pre	CellSearch	EpCAM, CK (8, 18, 19)	≥ 1 cell	21/53 (40)	-	-	-	Estimated from K-M curve (10)	PFS, OS	No
			ISET	cell size and CD45	≥ 1 cell	24/27 (89)						
			IHC	CK (4, 5, 6, 7, 8, 10, 13, 18), EpCAM, e-cadherin, Vimentin	≥ 1 cell	4/13 (31)						
Klos [40]	PB	Pre	RT-PCR	h-TERT	3× > than average expression in controls	22/50 (44)	-	-	-	Estimated from K-M curve (10)	OS	No
Kurihara [41]	PB	Pre	CellSearch	EpCAM, CK (8, 18, 19)	≥ 1 cell	11/26 (42)	-	7.7 (0–16)	-	Estimated from K-M curve (10)	OS	No
Rehders [42]	BM	Pre	IHC	CK (7, 8, 18)	-	12/49 (24)	-	-	-	Unable to estimate – insufficient data	DFS, OS	No
Roder [43]	BM	Pre	IHC	CK (1, 2, 5, 6, 7, 8, 11, 14, 16, 17, 18)	≥ 1 cell	25/48 (52)	-	22.8 (3–48)	-	Estimated from K-M curve (10)	OS	No
Sergeant [44]	PB	Pre, post	RT-PCR	EpCAM	-	10/40 (25)	-	-	24 (0.7–41.3)	Estimated from K-M curve (10)	DFS, OS	No
Soeth [45]	PB	Pre	RT-PCR	CK-20	Positive signal	52/154	81/172	-	-	Estimated from K-M curve (10)	OS	No
	BM					45/135						
van Heek [46]	BM	Pre	IHC	CK (8, 18)	≥ 1 cell	10/31	-	-	17 (2–24)	Estimated from K-M curve (10)	OS	No
Vogel [47]	BM	Pre	IHC	C54–0 (epithelial membrane antigen), CK (1, 2, 5, 6, 7, 8, 11, 14, 16, 17 ,18), mucin, CEA, Ca-19–9, 17–1A (membrane antigen)	≥ 1 cell	27/71	-	-	10.7 (2–61)	Estimated from K-M curve (10)	OS	No
Zhang [48]	PB	Pre	IHC and FISH	CK, CD25, DAPI and CEP8	≥ 2 cell	15/22	-	-	-	Unable to estimate – insufficient data	OS	No

*PB* peripheral blood, *BM* bone marrow, *ICC* immunocytochemistry, *IHC* immunohistochemistry, *RT-PCR* reverse transcription polymerase chain reaction, *OS* overall survival, *DFS* disease-free survival, *PFS* progression-free survival, *SD* standard deviation

**Table 3:** Risk assessment of the included studies of this systematic review

First author	Were adequate eligibility criteria developed and applied?	Was the measurement of both exposure and outcomes adequate?	Was confounding adequately controlled for?	Was the follow-up complete and adequate in duration?	Are reports of the study free of suggestion of selective outcome reporting?	Was the study free of other problems that put it at a high risk of bias?	Risk of bias
Bidard [33]	Yes	Yes	Yes	Yes	Yes	Yes	Low
De Albuquerque [34]	Yes	Yes	Yes	Yes	Yes	Yes	Low
Effenberger [35]	Yes	Yes	Yes	Yes	Yes	Yes	Low
Hoffmann [36]	Yes	No	Yes	Unclear	Yes	Yes	High
Hu [37]	Yes	Yes	Yes	Yes	Yes	Yes	Low
Juhl [38]	Yes	Yes	Yes	Yes	Yes	Yes	Low
Khoja [39]	Yes	Yes	Yes	Yes	Yes	Yes	Low
Klos [40]	Yes	Yes	Yes	Yes	No	Yes	High
Kurihara [41]	Yes	Yes	Yes	Yes	Yes	Yes	Low
Rehders [42]	Yes	Yes	Yes	Yes	No	Yes	High
Roder [43]	Yes	Yes	No	No	No	Yes	High
Sergeant [44]	Yes	Yes	Yes	Yes	Yes	Yes	Low
Soeth [45]	Yes	Yes	Yes	Yes	Yes	Yes	Low
van Heek [46]	No	Yes	No	Yes	Yes	Yes	High
Vogel [47]	Yes	Yes	Yes	Yes	Yes	Yes	Low
Zhang [48]	Yes	No	Yes	No	No	Yes	High

### Meta-analysis

**Figure 4:**
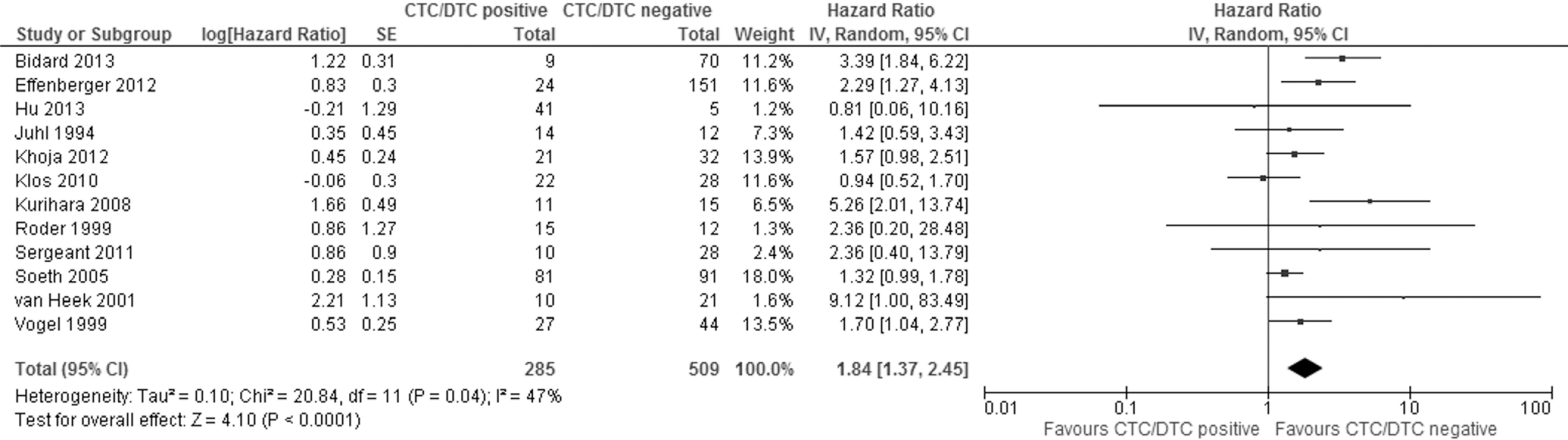
Forest plot of the hazard ratios of studies examining the relationship between CTC/DTC presence and overall survival

The meta-analysis of 12 studies examining the effect of CTCs/DTCs on OS demonstrated the detection of CTCs/DTCs corresponds with decreased OS (Figure 4 and Table 4). The combined HR was 1.84 (95% CI 1.37–2.45, *P* < 0.0001). There was moderate statistically significant heterogeneity between the studies based upon the I^2^ and Cochran’s Q statistic test (47% and *P* = 0.04, respectively). Ten studies demonstrated a significant association of CTC/DTC positivity with worse OS prognosis, while two studies showed HRs less than 1.

**Table 4:** Subgroup meta-analyses of overall and disease-/progression-free survival

Subgroups	OS						DFS/PFS					
	HR	95% CI	*I*^2^ (%)	*P* value	No. of studies	Subgroup heterogeneity *P* value (*I*^2^)	HR	95% CI	I2 (%)	*P* value	No. of studies	Subgroup heterogeneity *P* value (*I*^2^)
**Total**	1.84	1.37–2.45	47	< 0.0001	12	-	1.93	1.19–3.11	54	0.007	4	-
**Sampling site**						0.86 (0%)						0.57 (0%)
PB	2.03	1.14–3.63	65	0.02	6		1.95	0.97–3.92	67	0.06	3	
BM	1.91	1.36–2.68	0	0.0002	5		2.46	1.63–3.71	-	< 0.0001	1	
**Detection method**						0.03 (70.9%)						0.24 (30.5%)
CellSearch	2.79	1.39–5.63	71	0.004	3		1.34	0.94–1.90	-	0.11	1	
RT-PCR	1.25	0.96–1.62	0	0.09	4		2.67	1.11–6.39	47	0.03	2	
ID	1.91	1.36–2.68	0	0.0002	5		2.05	1.08–3.94	-	0.03	1	
**Treatment intent**						0.11 (55.6%)						0.15 (51.6%)
Potentially curable	0.94	0.52–1.70	-	0.84	1		-	-	-	-	-	
Palliative	2.25	1.06–4.77	74	0.04	2		1.34	0.94–1.90	-	0.11	1	
Both	1.80	1.35–2.42	22	<0.0001	9		2.67	1.11–6.39	47	0.03	2	
**Location of study**						0.51 (0%)						-
European	1.70	1.30–2.22	39	0.0001	10		1.93	1.19–3.11	54	0.007	4	
Non-European	3.02	0.57–16.11	46	0.20	2		-	-	-	-	-	
**Risk of bias**						0.98 (0%)						-
Low	1.93	1.43–2.59	46	< 0.0001	9		1.93	1.19–3.11	54	0.007	4	
High	1.97	0.49–7.92	51	0.34	3		-	-	-	-	-	
**CTC/DTC positive**						0.01 (84.6%)						0.84 (0%)
≥ 35 % of patients	1.51	1.15–1.99	31	0.003	8		2.16	0.75–6.19	84	0.15	2	
< 35 % of patients	2.86	1.91–4.28	0	< 0.00001	4		1.91	1.11–3.30	0	0.02	2	

**Figure 5:**
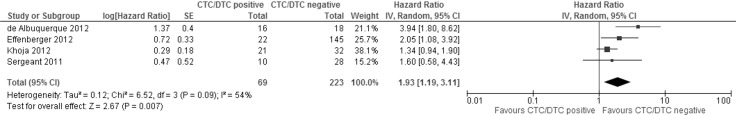
Forest plot of the hazard ratios of studies examining the relationship between CTC/DTC presence and disease-/progression-free survival

The meta-analysis of four studies examining the effect of CTC/DTC-positivity on DFS/PFS demonstrated that CTC/DTC-positivity was associated with shorter DFS/PFS (HR = 1.93, 95% CI 1.19 – 3.11, *P* = 0.007) (Figure 5 and Table 4). Moderate statistically significant heterogeneity was observed between the study results (I^2^ = 54%, *P* = 0.09). All the studies examined demonstrated decreased DFS/PFS in the CTC/DTC-positive patients, only varying with the degree of the effect seen.

### Sub-group analysis

Sub-group analyses were performed to examine the effect of sampling site, detection methods, risk of bias and the degree of CTC/DTC positive patients on the correlation between DFS/PFS and OS and CTC/DTC detection (Table 4).

#### Sampling site (CTCs vs. DTCs)

The presence of CTCs (via the detection from PB) and DTCs (via the detection of cells in the BM) both significantly corresponded with decreased OS (HR = 2.03, 95% CI 1.14 – 3.63, *P* = 0.02 and HR = 1.91, 95% CI 1.36 – 2.68, *P* = 0.0002, respectively). There was significant heterogeneity among the PB studies, while the BM study results were homogeneous. The subgroup analysis examining the relationship between CTC detection (PB sampling) and DFS/PFS found that patients positive for CTCs within the PB corresponded with decreased DFS/PFS (HR = 1.95, 95% CI 0.97 – 3.92, *P* = 0.06), but this was not significant. A subgroup analysis of the relationship between DTC detection within the BM and DFS/PFS was not possible because only one study presented the relevant data [35] which suggested worse PFS for DTC positive patients (HR = 2.46, 95% CI 1.63 – 3.71, *P* < 0.0001).

#### CTC/DTC detection methods

Three detection methods were used in the studies examined (CellSearch, RT-PCR and ID). Positive detection of CTCs by the CellSearch method had a significant correlation with decreased OS (HR = 2.79, 95% CI 1.39 – 5.63, *P* = 0.004). ID also had a significant relationship with decreased OS (HR = 1.91, 95% CI 1.36 – 2.68, *P* = 0.0002). There was a trend towards decreased OS in patients with RT-PCR detectable CTCs/DTCs (HR = 1.25, 95% CI 0.96 – 1.62), but this was not statistically significant (*P* = 0.09). RT-PCR significantly correlated CTC/DTC-positive patients with worse DFS/PFS (HR = 2.67, 95% CI 1.11 – 6.39, *P* =0.03). Only single studies compared CellSearch and ID CTC/DTC detection and PFS. CellSearch demonstrated a non-significant trend linking CTC/DTC-positivity with worse DFS/PFS (HR = 1.34, 95% CI 0.94 – 1.90, *P* = 0.11) [39], while ID detection showed a significant relationship between DTC-positivity and worse PFS (HR = 2.05, 95% CI 1.08 – 3.94, *P* = 0.03) [35].

#### Risk of bias

Studies determined to have low likelihoods of bias (Table 3) were analyzed and determined to demonstrate statistically significant correlations between OS and DFS/PFS and CTC/DTC detection (HR = 1.93, 95% CI 1.43 – 2.59, *P* < 0.0001 and HR = 1.93, 95% CI 1.19 – 3.11, *P* = 0.007, respectively, Table 4).

#### Prevalence of CTC/DTC-positive patients

Studies in which CTCs/DTCs were detected in less than 35% of patients demonstrated higher HRs for OS when compared with studies with detection rates equal to or greater than 35% (HR of 2.86 and 1.51, respectively, Table 4), yet the relationship between CTC/DTC detection and decreased OS was significant for both (*P* < 0.00001 and 0.003, respectively). Two studies examining DFS/PFS had CTC/DTC detection rates greater than or equal to 35%. Patients in these studies with detectable CTCs/DTCs had faster PC progression than those without detectable CTCs/DTCs (HR = 2.16), although this finding was not significant (*P* = 0.15).

### Studies not included in the meta-analysis

The three studies [36, 42, 48] which were not included in the pooled data presented mixed results regarding the significance of CTC/DTC detection, with Hoffman *et al.* [36] and Rehders *et al.* [42] finding non-significant relationships between CTC/DTC detection and survival, while Zhang *et al.* [48] found a significant association between CTCs and shorter OS.

## DISCUSSION

This study is the first systematic review and meta-analysis examining the prognostic value of both DTCs and CTCs in the setting of PC. The search identified 16 studies eligible for analysis, 13 of which were included in a pooled meta-analysis to elucidate the prognostic value of CTCs/DTCs in PC. The pooled data meta-analysis revealed CTC/DTC-positive patients had significantly worse OS (HR = 1.84, 95% CI 1.37–2.45, *P* < 0.0001) and significantly worse DFS/PFS (HR = 1.93, 95% CI 1.19–3.11, *P* = 0.007). The association with shorter OS was maintained when CTC positivity and DTC positivity were analyzed separately. Subgroup analysis of studies with only low risk of bias showed a significant association between OS/DFS/PFS and CTC/DTC positivity. This systematic review supports the findings of two previous meta-analyses regarding the prognostic role of CTCs in PC [31, 32] while expanding the breadth of data to include DTCs present within bone marrow aspirates. Additionally, we identified nine unique studies which had not previously been included in meta-analyses examining the prognostic value of CTCs in PC.

Subgroup analysis based on CTC/DTC detection methodology revealed that CTC/DTC positivity as detected by immunodetection and CellSearch both had a significant association with shorter OS. CTCs/DTCs detected via RT-PCR demonstrated a non-significant trend towards decreased OS. This may reflect the heterogeneity in the target genes used to identify CTCs/DTCs over time.

DTCs within the BM are thought to be clinically important because they may act as a reservoir for cancer cells allowing them to recirculate and establish metastases at distant sites. Pantel and Alix-Panabieres [49] suggested DTCs home to the BM where they survive within the hypoxic hematopoietic stem cell niche in a dormant state. This dormant state may provide protection against immune destruction and protection from chemotherapy agents. Likewise, DTCs may undergo protein expression changes, allowing them to survive in hostile environments and evade the immune system in addition to expressing proteins for increased motility and invasiveness [7]. Therefore, sampling of the BM, and the subsequent identification of DTCs, may be predictive of the metastatic potential of the disease at the time of diagnosis which can help to stratify patients to specific treatment regimes. Several meta-analyses have analyzed the prognostic value of DTCs and CTCs both in combination and individually in a variety of other solid cancers (colorectal [29, 30], gastric [22], ovarian [17], prostate [19]). Generally, the pooled results combining both DTC and CTC data corresponds with significantly worse DFS/PFS and OS, yet when DTCs and CTCs were analyzed separately only CTCs were significantly associated with poorer PFS and OS. The present study is the first to analyze the prognostic value of both DTCs and CTCs in PC, finding similar results to those studies mentioned above, indicating significantly worse PFS and OS in CTC/DTC positive patients. Unlike the above mentioned studies, where only CTCs were significantly associated with worse outcomes, the sub-group analysis from the present study indicated both DTCs and CTCs had similar prognostic significance for OS (HRs of 1.91 and 2.03, respectively). In the meta-analysis, only one study analyzed DTCs and PFS, which yielded a higher HR than the CTC subgroup (2.46 versus 1.95, respectively). These results suggest DTCs are of similar prognostic value to CTCs in PC and therefore either sampling site could be used for predicting patient outcomes at PC diagnosis.

PB sampling is more convenient for the patient, only requiring collection of a blood sample which can be performed in clinic and allows easy longitudinal follow-up. BM biopsies require more resources and expertise, are time consuming, painful for the patient, and have greater risk of serious complications such as hemorrhage and infection.

Blood samples from the portal venous system have been examined for the presence of CTCs in recent years [50–55]. Sampling from the portal vein is complicated, carries risks of complication and is invasive, requiring either endoscopic ultrasound guided fine needle aspiration [53] or sample collection during surgery [50–52, 54–56]. The CTC yield from portal venous samples is typically higher than the yield from PB samples [50, 53, 54]. Portal venous CTC-positivity has been demonstrated to be linked with increased rates of liver metastases [50, 54], worse PFS [51] and worse OS [55]. To date, the analysis of portal venous CTCs has only been demonstrated in isolated studies [50–56] and the prognostic utility of CTCs from portal venous blood has yet to be validated in larger cohorts.

In the present meta-analysis, the studies were subgrouped based on CTC/DTC detection methods (CellSearch, RT-PCR and ID), noting the studies were performed over a two decade period and there was wide heterogeneity among the detection methods. The RT-PCR sub-group analysis included studies utilizing a variety of target genes published over an eight-year period (Table 2), yet surprisingly the heterogeneity of the studies was extremely low (*I*^2^ = 0%) indicating all studies uniformly suggested CTC/DTC detection corresponded with worse survival. The studies using ID methodologies were similarly homogeneous (*I*^2^ = 0) with all studies uniformly demonstrating worse survival with CTC/DTC positivity. Surprisingly, the standardized detection method, CellSearch, produced a high level of interstudy heterogeneity for OS (*I*^2^ = 71%), but still suggested CTC/DTC detection corresponded with worse survival.

While none of the included studies examined the presence of CTCs/DTCs in pre-cancerous lesions such as pancreatic intraepithelial neoplasia (PanIN) this would be an area of great interest for future studies. If CTCs are released early in PC development it could change the understanding of the pathophysiology of PanIN and would have wide implications for management of this lesion.

Several studies have examined CTC detection as a diagnostic tool for PC [48, 57, 58] finding CTC detection to have high specificity (94–100%) but lower sensitivity (55–75%). The lack of diagnostic sensitivity, need for real-time processing of samples, and high cost prevents CTC/DTC detection from being a routinely performed investigation as part of the work-up for PC. However, in the future CTC/DTCs may find their utility in the realm of personalized medicine for cancer therapy, as advances are made in whole exome and targeted next generation sequencing of circulating tumor cells [59]. This would facilitate a minimally invasive liquid biopsy allowing for rapid detection of targetable driver mutations.

Along with CTC/DTC analysis several other blood-based molecular methodologies have been used for prognostication in PC such as circulating tumor DNA (ctDNA). The *KRAS* gene is frequently used to identify ctDNA released from PC cells as > 90% of PCs have mutations within the *KRAS* gene [60, 61]. Multiple studies have utilized detection of the mutated *KRAS* gene within the ctDNA of PC patients [62–66], subsequently finding ctDNA *KRAS* mutations are present within 27 – 71% of PC patients. Several studies have reported significant associations between the detection of *KRAS*-mutant ctDNA and poor survival (PFS and OS) [62, 63, 65–67]. A recent systematic review and meta-analysis by Li *et al.* [68] found a significant association between *KRAS* mutations in PC liquid biopsies and worse OS (HR 3.16, 95% CI 2.1–4.71, *P* < 0.01). Another study by Earl *et al.* [69] published after the literature search was performed for the present study, analyzed both ctDNA and CTCs for their ability to be detected and their prognostic value in PC. *KRAS-*mutant ctDNA was detected in 18% of PC patients and ≥ 1 CTC was detected in 16% of PC patients, with OS being significantly worse for both ctDNA- and CTC-positive patients. In patients where both detection methods were used, 80% of CTC-positive patients were also *KRAS* ctDNA positive, while one *KRAS*-positive patient was CTC-negative. The CTC-positive patients had significantly shorter OS (HR = 3, 95% CI 1.16 – 7.38, *P* = 0.023) and the *KRAS*-mutant positive patients also had worse OS (HR = 12.2, 95% CI 3.6 – 40.7, *P* < 0.001). The use of ctDNA has the distinct advantage of being more robust than CTCs, therefore not requiring real-time processing. This allows for cold storage of plasma samples until the time of analysis.

Several studies have reported identifying clusters of CTCs (≥ 2 CTCs attached to each other), known as circulating tumor metastases (CTM) in PC [39, 70, 71]. Chang *et al.* [70] found patients with more CTM detected (≥ 30 CTM per 2 mL blood) had worse PFS and OS compared with those with lower levels of CTM. Hong *et al.* [72] published a review of CTM suggesting CTM tend to be polyclonal (containing a mixture of CTCs and other support cell types including mesenchymal cells, cancer-associated fibroblasts, pericytes, immune cells and platelets) which is thought to enhance the metastatic ability of CTCs within the CTMs.

Ting *et al.* [73] isolated individual CTCs from pancreatic cancer and performed single-cell RNA sequencing, finding high level expression of extracellular matrix proteins, including SPARC, which is involved in cell migration and invasiveness. Future studies on CTM identification and characterization may help in the diagnosis and prognosis of PC patients. Likewise, characterization of CTM may reveal not only potential drug targets in CTCs, but also in non-malignant supportive cells which may promote metastatic spread.

Very few studies have addressed the presence of CTCs/DTCs before and after neo-adjuvant therapy in PC. Poruk *et al.* [56] found there was no significant difference in CTC positivity between patients who received neoadjuvant therapy and those who did not. Additionally, there was little association with the presence of CTCs and PC recurrence after surgery. Kulemann *et al.* [74] noted neoadjuvant therapy did not appear to influence the presence of CTCs as 83% (5/6) of the patients analyzed had CTCs present following neo-adjuvant therapy. Ren *et al.* [75] examined blood samples of 41 late stage (III or IV) PC patients before and after commencing 5-fluorouracil (5-FU) treatment, finding prior to treatment 80.5% of patients had > 2 CTCs in 7.5mL of PB, but after seven days of 5-FU this had reduced to 29.3% of patients. Unfortunately, the significance of this on survival outcomes was not presented. Further studies with larger patient cohorts are required to determine the significance of CTC/DTC detection before and after neo-adjuvant therapy and the relationship with patient prognosis.

Within this study there was moderate heterogeneity between studies for the pooled analysis due to the diverse patient groups examined and variety of methodologies used. Therefore, we employed a random effects models were used to provide more conservative estimates of the effect of CTC/DTC detection on prognosis. The small patient cohorts in each of the analyzed studies and relatively small number of studies, particularly for the DTC analyses, may have distorted the meta-analysis results. Global standardization of CTC/DTC detection techniques may help to reduce or remove some heterogeneity among studies. In future studies subgroup analyses investigating patient CTC/DTC status with TNM staging, number of CTCs/DTCs detected, treatment (including neoadjuvant therapy) and their relationships with survival may provide greater insight regarding the utility of CTC/DTC detection in the staging and prognosis of PC patients.

## MATERIALS AND METHODS

### Search strategy

Throughout the development and implementation of this study the recommendations of the Preferred Reporting Items for Systematic Reviews and Meta-Analyses (PRISMA) statement [76] and the Meta-analysis Of Observational Studies in Epidemiology (MOOSE) checklist [77] were applied.

### Literature search

The following databases were systematically searched on July 27th 2015: Medline, Pre-Medline, Embase, SCOPUS, Web of Science, Science Citation index, BIOSIS previews, Pubmed, Cinahl, Cochrane library, ClinicalTrials.gov, Australian New Zealand Clinical Trials Registry, and World Health Organization International Clinical Trials Registry Platform. Search strings for each database are described in the Supplementary Information. No language restriction, date restriction, or publication status restriction were used. The reference lists of all included articles were hand checked for additional relevant articles not identified in the database searches. Full-text articles were retrieved for any articles deemed potentially eligible. Additionally, cited reference searches were performed on the articles identified as relevant full-text articles using Science Citation Index and Web of Science databases to identify any additional relevant articles.

### Eligibility criteria

Prospective or retrospective studies comparing OS, DFS or PFS in CTC/DTC positive patients compared with CTC/DTC negative patients were deemed appropriate for this review and meta-analysis. Conference abstracts and letters were excluded. The participants in the included studies were ≥ 18 years of age with histologically proven PC. Samples for the analysis of CTC/DTCs were collected from the peripheral blood (PB) and/or BM of the PC patients. All methods of CTC/DTC detection were included for analysis. Studies with less than 20 participants were excluded. Author lists, institutions and patient recruitment dates were examined to avoid including duplicate data. Studies with insufficient data to calculate the hazard ratio (HR) for either DFS/PFS and/or OS were included in the systematic review but not the meta-analysis.

### Data extraction

Two review authors (DS, CN) independently assessed references identified by the searches and evaluated them against the inclusion criteria. Disagreements on the selection of relevant studies were resolved by the discussion among the authors. The following data were extracted from each of the included studies: study characteristics (first author, year of publication, country of origin, patient characteristics [number of participants, sex, age, cancer staging information], treatment intent, treatment received), study design (sampling site, sampling time, CTC/DTC detection method, target gene/antigen, duration of follow-up), and study outcomes (baseline and overall CTC/DTC positive rate, survival outcomes, univariate and/or multivariate analyses).

### Risk of bias analysis

The quality of the studies and their potential to introduce bias was assessed using a modified version of the Cochrane Collaboration’s risk of bias tool as described by Rahbari *et al.* [30]. Publication bias was assessed using funnel plots which present the effect measured by the inverse of the studies’ standard error.

### Statistical analyses

The HR was used to evaluate the impact of CTC status on progression and survival. HR and associated standard error data were extracted from studies where they were presented. If the HR value and corresponding standard error data was not presented in the published article, but sufficient data was available within the text, the methods of Tierney *et al.* [78] were used to estimate the HR value, confidence interval (CI) and *P* values. HRs were calculated so values > 1 denote a worse prognosis in the tumor-positive group. The HR values of each study were pooled together using the generic inverse variance method within Review Manager (RevMan) Version 5.3 (Copenhagen: The Nordic Cochrane Centre, The Cochrane Collaboration, 2014) [79]. *P* values ≤ 0.05 were considered statistically significant. Heterogeneity was assessed using the Cochran’s *Q* statistical test and the *I*^2^ value [80]. *P* values ≤ 0.1 and/or *I*^2^ percentages > 50% were considered to reflect significant heterogeneity. Summary HR values were calculated using a random-effects analysis model. Sub-group analyses were performed where two or more studies examined the same variables along with CTC/DTC status and DFS/PFS or OS.

## CONCLUSIONS

This is the first meta-analysis to analyze the prognostic value of both CTCs and DTCs in PC. The results indicate that regardless of the sampling site or detection method, patients positive for the presence of CTCs/DTCs have worse prognosis. Standardization of sampling and detection methods may add to the clinical utility of CTC identification and disease prognosis, which may then allow CTCs/DTCs to be used as part of the staging process and for patient treatment stratification.

## SUPPLEMENTARY MATERIALS



## Abbreviations

BM: Bone marrow
CI: Confidence interval
CTC: Circulating tumor cell
CtDNA: Circulating tumor deoxyribonucleic acid
CTM: Circulating tumor metastases
DFS: Disease free survival
DTC: Disseminated tumor cell
HR: Hazard ratio
ICC: Immunocytochemistry
ID: Immunodetection
IHC: Immunohistochemistry
OS: Overall survival
PanIN: Pancreatic intraepithelial neoplasia
PB: Peripheral blood
PC: Pancreatic cancer
PFS: Progression free survival
RT-PCR: Reverse transcriptase polymerase chain reaction
