# Supplementary material for: Circulating and disseminated tumor cells in pancreatic cancer and their role in patient prognosis: a systematic review and meta-analysis
Source: Oncotarget. 2017 Aug 4;8(63):107223–36. doi: 10.18632/oncotarget.19928 (PMC5739809; doi:10.18632/oncotarget.19928)
Supplement: Supplementary file 1 [file oncotarget-08-107223-s001.pdf]

Circulating and disseminated tumor cells in pancreatic cancer and their role in patient prognosis: a systematic review and meta-analysis

SUPPLEMENTARY MATERIALS

Medline Search

No.	Search Term	Results
1	exp Pancreatic Neoplasms/	59146
2	(pancrea* adj5 (cancer* or carcinom* or neoplas* or tumo* or metast* or malignan* or adenocarcinoma* or cystadenocarcinoma*)).mp.	70427
3	1 or 2	72447
4	neoplasm micrometastasis/	10591
5	neoplasm seeding/	382
6	neoplastic cells, circulating/	7418
7	Neoplasm, Residual/bl, sc [Blood, Secondary]	116
8	bone marrow examination/	6047
9	bone marrow neoplasms/sc	921
10	((dis?eminat* or des?iminat* or circulat* or isolat*) adj3 (tumo* cell* or cancer* cell* or neoplas* cell* or carcinoma cell* or malignan* cell* or metast*)).mp.	17076
11	4 OR 5 OR 6 OR 7 OR 8 OR 9 OR 10	26769
12	(hematogen* or haematogen* or blood or “bone marrow”).mp.	2172550
13	11 AND 12	12347
14	3 AND 13	182
15	exp prognosis/	1188069
16	exp survival analysis/	204306
17	exp epidemiologic studies/	1795970
18	(prognos* or survival* or recurren* or relaps* or predict* or mortal* or inciden*).mp.	3187290
19	15 OR 16 OR 17 OR 18	4527848
20	14 AND 19	117

Pre-MEDLINE

No.	Search Term	Results
1	(pancrea* adj5 (cancer* or carcinom* or neoplas* or tumo* or metast* or malignan* or adenocarcinoma* or cystadenocarcinoma*)).mp.	4850
2	((dis?eminat* or des?iminat* or circulat* or isolat*) adj3 (tumo* cell* or cancer* cell* or neoplas* cell* or carcinoma cell* or malignan* cell* or metast*)).mp.	1322
3	(hematogen* or haematogen* or blood or bone marrow).mp.	96240
4	2 AND 3	474
5	1 AND 4	14
6	(prognos* or survival* or recurren* or relaps* or predict* or mortal* or inciden*).mp.	294944
7	5 AND 6	7

EMBASE

No.	Search Term	Results
1	‘pancreas tumor’/exp AND [embase]/lim	84613
2	pancrea* NEAR/5 (cancer* OR carcinom* OR neoplas* OR tumo* OR metast* OR malignan* OR adenocarcinoma* OR cystadenocarcinoma*) AND [embase]/lim	95188
3	1 OR 2	102340
4	‘tumor embolism’/exp AND [embase]/lim	984
5	‘tumor seeding’/exp AND [embase]/lim	756
6	‘micrometastasis’/exp AND [embase]/lim	4012
7	‘bone marrow examination’/exp AND [embase]/lim	29577
8	‘bone marrow metastasis’/exp AND [embase]/lim	2115
9	(dis?eminat* OR des?iminat* OR circulat* OR isolat*) NEAR/3 (tumo* OR cancer* OR neoplas* OR carcinoma OR malignan* OR metast*) NEAR/1 cell* AND [embase]/lim	13477
10	#4 OR #5 OR #6 OR #7 OR #8 OR #9	49038
11	(hematogen* OR haematogen* OR blood OR ‘bone marrow’) AND [embase]/lim	3308831
12	#10 AND #11	37834
13	#3 AND #12	437
14	‘prognosis’/exp AND [embase]/lim	396190
15	‘survival’/exp AND [embase]/lim	649351
16	‘epidemiology’/exp AND [embase]/lim	1861210
17	prognos* OR survival* OR recurren* OR relaps* OR predict* OR mortal* OR inciden* AND [embase]/lim	3724031
18	#14 OR #15 OR #16 OR #17	4464715
19	#13 AND #18	262

Science Citation Index

No.	Search Term	Results
1	TS= Pancreatic Neoplasms/	7283
2	TS=(pancrea* NEAR/5 (cancer* OR carcinom* OR neoplas* OR tumo* OR metast* OR malignan* OR adenocarcinoma* OR cystadenocarcinoma*))	70703
3	#1 OR #2	71238
4	TS= Neoplasm micrometastasis/	112
5	TS= Neoplasm, Residual	2242
6	TS= Bone marrow examination/	7073
7	TS= Neoplasm seeding/	539
8	TS= Neoplastic cells, circulating/	673
9	TS=((dis?eminat* OR des?iminat* OR circulat* OR isolat*) NEAR/3 (“tumo* cell*” OR “cancer* cell*” OR “neoplas* cell*” OR “carcinoma cell*” OR “malignan* cell*” OR metast*))	15548
10	#4 OR #5 OR #6 OR #7 OR #8 OR #9	25839
11	TS=(hematogen* OR haematogen* OR blood OR “bone marrow”)	1620849
12	#10 AND #11	12434
13	#3 AND #12	240
14	TS= prognosis/	250270
15	TS= survival analysis/	208443
16	TS= epidemiologic studies/	38412
17	TS=(prognos* OR survival* OR recurren* OR relaps* OR predict* OR mortal* OR inciden*)	4271779
18	#14 OR #15 OR #16 OR #17	4295324
19	#13 AND #18	150

SCOPUS

No.	Search Term	Results
1	TITLE-ABS-KEY ((pancrea* W/5 (cancer* OR carcinom* OR neoplas* OR tumo* OR metast* OR malignan* OR adenocarcinoma* OR cystadenocarcinoma*)))	109511
2	TITLE-ABS-KEY (((dis?eminat* OR des?iminat* OR circulat* OR isolat*) W/3 ("tumo* cell*" OR "cancer* cell*" OR "neoplas* cell*" OR "carcinoma cell*" OR "malignan* cell*" OR metast*)))	21532
3	TITLE-ABS-KEY (hematogen* OR haematogen* OR blood OR "bone marrow")	4070001
4	#2 AND #3	7494
5	#1 AND #4	284
6	TITLE-ABS-KEY ((prognos* OR survival* OR recurren* OR relaps* OR predict* OR mortal* OR inciden*))	6291717
7	#5 AND #6	173

Cochrane Library

No.	Search Term	Results
1	MeSH descriptor: [Pancreatic Neoplasms] explode all trees	967
2	(pancrea* near/5 (cancer* or carcinom* or neoplas* or tumo* or metast* or malignan* or adenocarcinoma* or cystadenocarcinoma*))	2258
3	#1 OR #2	2266
4	MeSH descriptor: [Neoplasm Micrometastasis] explode all trees	12
5	MeSH descriptor: [Neoplasm Seeding] explode all trees	31
6	MeSH descriptor: [Neoplastic Cells, Circulating] explode all trees	85
7	MeSH descriptor: [Neoplasm, Residual] explode all trees and with qualifier(s): [Blood - BL, Secondary - SC]	1
8	MeSH descriptor: [Bone Marrow Examination] explode all trees	66
9	MeSH descriptor: [Bone Marrow Neoplasms] explode all trees and with qualifier(s): [Secondary - SC]	9
10	((dis?eminat* or des?iminat* or circulat* or isolat*) near/3 (tumo* cell* or cancer* cell* or neoplas* cell* or carcinoma cell* or malignan* cell* or metast*))	424
11	#4 OR #5 OR #6 OR #7 OR #8 OR #9 OR #10	538
12	(hematogen* or haematogen* or blood or "bone marrow")	216774
13	#11 AND #12	260
14	#3 AND #13	10
15	MeSH descriptor: [Prognosis] explode all trees	112396
16	MeSH descriptor: [Survival Analysis] explode all trees	16084
17	MeSH descriptor: [Epidemiologic Studies] explode all trees	119402
18	(prognos* or survival* or recurren* or relaps* or predict* or mortal* or inciden*)	207296
19	#15 or #16 or #17 or #18	321193
20	#14 and #19	10

BIOSIS Previews

No.	Search Term	Results
1	TS= Pancreatic Neoplasms/	54613
2	TS=(pancrea* NEAR/5 (cancer* OR carcinom* OR neoplas* OR tumo* OR metast* OR malignan* OR adenocarcinoma* OR cystadenocarcinoma*))	70027
3	#1 OR #2	74836

4	TS= Neoplasm micrometastasis/	1633
5	TS= Neoplasm, Residual	27614
6	TS= Bone marrow examination/	8717
7	TS= Neoplasm seeding/	8598
8	TS= Neoplastic cells, circulating/	14060
9	TS=((dis?eminat* OR des?iminat* OR circulat* OR isolat*) NEAR/3 (“tumo* cell*” OR “cancer* cell*” OR “neoplas* cell*” OR “carcinoma cell*” OR “malignan* cell*” OR metast*))	12074
10	#4 OR #5 OR #6 OR #7 OR #8 OR #9	67957
11	TS=(hematogen* OR haematogen* OR blood OR “bone marrow”)	5679458
12	#10 AND #11	42219
13	#3 AND #12	853
14	TS= prognosis/	223689
15	TS= survival analysis/	152490
16	TS= epidemiologic studies/	34668
17	TS=(prognos* OR survival* OR recurren* OR relaps* OR predict* OR mortal* OR inciden*)	2545288
18	#14 OR #15 OR #16 OR #17	2568879
19	#13 AND #18	388
